# How work and family caregiving responsibilities interplay and affect registered dietitian nutritionists and their work: A national survey

**DOI:** 10.1371/journal.pone.0248109

**Published:** 2021-03-10

**Authors:** Karla Williams, Dennis Eggett, Emily Vaterlaus Patten

**Affiliations:** 1 Department of Nutrition, Dietetics, and Food Science, Brigham Young University, Provo, Utah, United States of America; 2 Department of Statistics, Brigham Young University, Provo, Utah, United States of America; Universitat de Valencia, SPAIN

## Abstract

Healthcare professionals provide paid care at work and potentially have caregiving responsibilities outside of work; work responsibilities in addition to child and/or elder care is considered double- or triple-duty care. Employees may experience conflict and/or enrichment as their work and family responsibilities interface. This study’s purpose is to explore the work and family interface of Registered Dietitian Nutritionists (RDNs), determine the prevalence of work-family conflict and enrichment, and identify characteristics associated with higher work-family conflict and enrichment scores. A survey instrument assessing caregiving responsibilities and work-family conflict and enrichment was distributed electronically to 4,900 RDNs throughout the United States. Frequencies, means, correlative relationships, and ANCOVA were calculated using SAS software 9.04. Of 1,233 usable responses, nearly two-thirds of RDNs (65.5%) reported providing either double-duty or triple-duty care. About half of RDNs (47.2%) reported work-family conflict and fewer (14.8%) reported family-work conflict. Additionally, most RDNs (79.4%) reported work-family enrichment and even more (85.2%) reported family-work enrichment. Higher work-family conflict scores had correlative relationships with higher levels of burnout, lower life satisfaction, and higher intent to quit. Higher work-family enrichment scores had correlative relationships with lower burnout, higher job satisfaction, higher career satisfaction, higher life satisfaction, and lower intent to quit. Understanding the unpaid caregiving responsibilities of RDNs and the interface of work/family responsibilities may provide insight into career planning for RDNs and guide managers of RDNs in efforts to amplify the contribution of RDNs.

## Introduction

Dietetics professionals apply the science of food and nutrition as they work with individuals, groups, communities and populations to promote health and prevent/treat disease [1]. Fifty-nine percent RDNs provide clinical care in acute, long-term, and ambulatory/outpatient settings [2]. Clinical nutrition involves using the Nutrition Care Process including medical nutrition therapy in working with patients/clients with diseases or conditions that nutrition plays a role [3]. Clinical RDNs conduct assessments and reassessments of patients/clients (nutrition-focused physical examinations and interviews), provide education, participate in rounds/team meetings, and indirect care activities (e.g., presentations, meetings, administrative work, and mentoring students) [4,5]. RDNs who do not provide clinical care often work in the community, food and nutrition management, consultation and business, or education and research and their work roles vary [2].

In the USA, there were nearly 71,000 jobs for RDNs and nutritionists in 2018, and the job outlook is growing much faster (11%) than the average of other jobs (5%) from 2018 to 2028 [6]. The Academy of Nutrition and Dietetics’ Workforce Demand Study indicated that demand for dietetic services will/would exceed the supply of credentialed practitioners by 2020 [7]. Globally and specifically within the United States, dietetics remains a predominantly female profession with female representation greater than 90% [2,8–11]. As leaders of the profession continue efforts to diversify the gender profile of the profession, they should simultaneously attend to its current workforce’s unique circumstances to encourage retention and success.

Across both developing and developed countries, women spend more time than men working when unpaid and paid work are combined [12]. On average, women spend two (developed countries) to three (developing countries) more hours per day than men on unpaid work which includes domestic (e.g, child care, elder care, food preparation, etc.) and volunteer activities [12]. In the United States, highly educated women are more likely to become mothers than they were ten years ago, and women are now becoming mothers later in life [13] suggesting potential child care responsibilities for RDNs. Furthermore, the typical informal caregiver for older adults in the United States is a 49-year-old woman who works outside of the home [14]. Research in the nursing profession has recognized healthcare practitioners provide formal, nonfamily paid care at work and informal, unpaid care outside of work (e.g., child and/or elder care). Caregivers have been categorized into four types: nonfamily caregivers (provide care solely at work), double-duty child caregivers (provide care at work and unpaid child care outside of work) [15], double-duty elder caregivers (provide care at work and unpaid elder care outside of work), and triple-duty caregivers (provide care at work and both child care and elder care outside of work [16].

Furthermore, as RDNs attend to their work and family responsibilities, they may experience conflict. Work-family conflict (WFC) and family-work conflict (FWC) are forms of inter-role conflict in which the demands, time requirements, and strain of work or family responsibilities are incompatible at some level [17,18]. Employees who experienced this conflict had poorer work performance [17]. The prevalence of WFC and FWC has been assessed in other healthcare professions [19–21] like nursing but not sufficiently within dietetics.

Work and family may enrich each other; work-family enrichment is bi-directional and assesses “the extent to which experiences in one role improve the quality of life in the other role” [22, p73]. When employees have enrichment between these roles, they likely have better relationships and performance in both [23]. Turnover intention is lower for those who experience enrichment from their work [24]. There is a positive relationship between work-family enrichment (WFE) and job satisfaction and affective commitment [25]. There is also a positive relationship between family-work enrichment (FWE) and family satisfaction [25].

Research exists about the work/life interface of dietitians in the USA, but it is limited. Several studies assess dietitian burnout, but they are dated and outside of the USA [26,27]. One recent domestic study examined regular physical activity, work-life balance, and family support as influential factors on life satisfaction of RDNs [28], and more recently a study of multiple practitioners across a variety of healthcare professions found RDNs experienced one of the highest levels of work-life integration [29]. However, it important that the interplay of work and family responsibilities of RDNs be fully explored because women are more likely than men to hold caregiving responsibilities outside of work [30–32], and due to the demographic profile of the profession, the dietetics workforce may be disproportionately affected by additional unpaid caregiving responsibilities.

This study used a cross-sectional survey to investigate the work/family interface of RDNs and may provide strategies for attracting and retaining qualified RDNs. The objectives of this study are to: 1) determine the prevalence of caregiving responsibilities among RDNs, 2) determine the prevalence of WFC/FWC and FWE/WFE for RDNs, 3) identify correlative relationships of higher WFC/FWC and FWE/WFE scores with various work characteristics, and 4) identify personal and family characteristics contributing to higher WFC/FWC and FWE/WFE scores.

## Materials and methods

### Study design, participants, and procedure

Brigham Young University’s Institutional Review Board approved this study (#17527) and participants implied consent by completing the survey instrument. Data concerning unpaid caregiving responsibilities, WFC/FWC, and WFE/FWE was collected though a national cross-sectional study design. Study participants were recruited via a randomly generated email list of 5,000 RDNs throughout the United States and its territories who had self-identified with the Commission on Dietetic Registration (CDR; the credentialing organization for the profession in the USA and its territories) as practicing in clinical dietetics. Survey participants who indicated they held a position in a non-clinical dietetics practice area at the time of the study (despite indicating practicing in clinical dietetics with CDR) were retained in the sample and categorized as such for analysis. As an incentive, participants were eligible to enter to win one of seventy-five $15 Amazon gift cards.

Data was collected online via Qualtrics software through a one-time, 51-item survey questionnaire comprised of several validated survey instruments measuring WFC/FWC, WFE/FWE, and family/work characteristics. Several supplemental survey questions were developed by researchers and also included. Those who were practicing at the time of the study received questions exploring WFC/FWC, WFE/FWE, and family/work characteristics. All protocols, materials, and communications were approved by the university’s institutional review board (IRB). Additional permission to access the study sample was obtained from CDR upon IRB approval. Participants indicated consent by completing the survey instrument.

An expert panel (n = 3) of dietetics, management, and family studies scholars evaluated each question within the survey instrument on three criteria: appropriateness of the question for this research, importance for this research, and phrasing (phrasing of previously validated measures was not changed) [33]. The Kansas Marital Satisfaction Survey (KMSS) to measure marriage distress was a recommended addition to the instrument from this review [34].

Cognitive interviews were conducted using Zoom videoconferencing technology and in-person interviews. Cognitive interview participants (n = 7) were from three states, ranged in age from 29–61 years, had varying caregiving responsibilities, and had between 5–26 years of dietetics experience. Changes derived from the cognitive interviews were reflected in the survey instrument and included adding a time frame of six months to several questions, the provision of instructions for reporting the age of the youngest child living at home, and—preceding questions exploring family relationships—a definition of family (“There are diverse types of families, please respond to the following questions based on what you consider to be your family”). A pilot test was conducted with a random sample of 100 of the 5,000 names CDR provided (n = 20); no changes were made based on the pilot study. Finally, the remaining study participants (n = 4,900) were contacted via email invitation in April 2018 to participate in the actual study and up to two reminder emails were sent. Completion of the survey instrument indicated implied consent.

### Measures

#### Caregiving responsibilities

Questions concerning child care responsibilities were adapted and modified from Grzywacz et al [21]. *Child caregiving* status was determined using the question “Are you a parent, step parent, or legal guardian of a child or children who live with you full-time or part-time?” If “yes,” they were asked how many children were living with them full or part-time, and the age of the youngest child living at home. *Elder care* responsibilities were defined as “providing unpaid care (e.g., managing a person’s finances, arranging for outside services, visiting regularly to see how they are doing, helping with personal needs, or household chores) for at least 3 hours per week to an older adult, regardless of their living arrangement” [14,16]. Finally, self-rated *strain of unpaid caregiving* was measured using the question “how burdensome do you feel your unpaid child or elder caregiving responsibilities (either family or non-family) have been over the past 6 months?” [35].

#### Work-family conflict

The WFC/FWC scale consisted of two validated subscales, one measuring work-to-family conflict (WFC) and another measuring family-to-work conflict (FWC) [18]. The total scale consisted of 10 statements using a 7-point Likert scale. Statement examples include: “The demands of my work interfere with my home and family life” (WFC) and “Things I want to do at work don’t get done because of the demands of my family or spouse/partner” (FWC). Scores for each subscale were averaged and higher scores indicated higher levels of WFC and FWC respectively.

#### Work-family enrichment

The WFE/FWE scale consisted of two validated subscales, one measuring work-to-family enrichment (WFE) and the other measuring family-to-work enrichment (FWE) [23]. The total scale consisted of six statements using a 5-point Likert scale. Examples of statements include: “My involvement in work helps me to understand different viewpoints and this helps me be a better family member” (WFE) and “My involvement in my family helps me acquire skills and this helps me be a better worker” (FWE). Scores for each subscale were averaged and higher scores indicated higher levels of enrichment.

#### Work characteristics

A series of measures assessing participants’ experiences and feelings about their work were administered. These included: burnout (one item) [36], job satisfaction (one item) [37,38], career satisfaction (one item) [35], intent to quit (two items) [38], and intent to leave the profession (one item) [39]. Additionally, information about participants’ current area of dietetics practice, number of jobs held, and hours working for pay each week was collected.

#### Personal and family characteristics

Demographic information such as age, gender, race, ethnicity, level of education, household income, and marital status were obtained. If a participant was married/partnered, the spouse/partner’s average hours working for pay each week was collected. Life (one item) [36] and marital (three items) [34] satisfaction were both assessed.

### Data analysis

All statistics were calculated using SAS software version 9.04. Frequencies and means were calculated for each variable. Correlative relationships between conflict and enrichment scores (WFC, FWC, WFE, and FWE) and various work characteristics (e.g., burnout and career satisfaction) were calculated. Analysis of covariance (ANCOVA) was used to compute statistical significance between the independent variables and the dependent variables (WFC, FWC, WFE, and FWE). ANCOVAs were computed separately for those with and without unpaid caregiving responsibilities due to branching questions that were not shown to all participants. Additionally, Tukey analyses were used to compare differences in conflict and enrichment between those who had caregiving responsibilities and those who did not. Different analyses were conducted on the eight different dependent variables, therefore, a Pseudo Bonferroni’s adjustment with a critical alpha level of .01 was applied. Of variables identified for use in this study, none were excluded from the analysis and all variables were controlled for. Finally, all post hoc values for pair wise analyses were Tukey adjusted.

## Results

In total, 1,118 usable responses were gathered with representation from all 50 U.S. states and Puerto Rico. Of participants, 92.2% (n = 1,031; Table 1) held a paid dietetics position and were presented items addressing work-family interface. Most indicated they were female (95.3%) and white (87.3%). The mean age was 46.6 ± 11.4 years. Of those practicing, 81.5% held a clinical dietetics position and 51.8% worked 36–45 hours/week. Most (79.7%) were married/partnered, and 23.2% were in a distressed marriage/partnership.

**Table 1:** Registered Dietitian Nutritionists’ (RDN) personal and family characteristics (n = 1,031).

	n	%		n	%
**Age**			**Number of paying jobs**		
≤ 34 years	176	18.0	1	683	71.7
35–44 years	281	28.7	2	199	20.9
45–54 years	216	22.1	3	71	7.5
55–64 years	265	27.1	**RDN work hours/week**		
≥65 years	41	4.2	≤15	50	5.7
**Gender**			16–25	102	11.6
Female	982	95.3	26–35	133	15.1
Male	20	1.9	36–45	457	51.8
Prefer not to answer	29	2.8	>45	141	16.0
**Race**			**Spouse/partner work hours/week**		
White	900	87.3	≤15	13	1.8
Asian	50	4.9	16–25	24	3.3
Other	65	6.3	26–35	37	5.1
Black or African American	16	1.6	36–45	388	53.5
**Hispanic/Latino**			>45	263	36.3
No	953	92.4	**Number of children at home**		
Yes	41	4.0	0	456	44.2
Prefer not to answer	37	3.6	1	205	19.9
**Marital Status**			2	272	26.4
Married/partnered	822	79.7	3	83	8.1
Single, never married	116	11.3	4+	15	1.5
Other	93	9.0	**Age of youngest child at home**		
**Marital/Partner Satisfaction**			No children at home	461	44.7
Not married	221	21.4	<6 years	220	21.3
Non-distressed	571	55.4	6–11 years	140	13.6
Distressed	239	23.2	12–17 years	104	10.1
**Household Income**			≥18 years	106	10.3
Less than $50,000	29	2.9	**Elder care**		
$50,000 - $59,9999	46	4.5	No	842	81.8
$60,000 - $69,9999	70	6.9	Yes	187	18.2
$70,000 - $79,9999	67	6.6	**Caregiving Status**		
$80,000 - $89,9999	60	5.9	Nonfamily	358	34.5
$90,000 - $99,9999	86	8.5	Double-duty: child	487	47.4
$100,000 - $149,999	324	32.0	Double-duty: elder	93	9.1
More than $150,000	202	19.9	Triple-duty	93	9.1
Prefer not to answer	130	12.8	**Self-Reported Strain of Unpaid Caregiving**		
**Primary Breadwinner**			<25	187	30.0
Spouse/partner	477	46.3	25–49.9	153	24.6
RDN	213	20.7	50–74.9	204	32.7
Equal contributors	94	9.1	75–100	79	12.7
Prefer not to answer	23	2.2			

### Caregiving and conflict/enrichment

Nearly half (47.4%) of participants had double-duty child care responsibilities, 9.1% had double-duty elder care responsibilities, and 9.1% had triple-duty caregiving responsibilities (both child and elder care). Individuals providing double-duty child care (*P* = 0.0042) and double-duty elder care (*P* = 0.0024) experienced higher levels of WFC than nonfamily caregivers. Those providing double-duty child care (*P*<0.0001), double-duty elder care (*P*<0.0001), and triple-duty care (P<0.0001) experienced higher FWC than nonfamily caregivers. No significant relationships between WFE and caregiving status were found. It is suggestive but not conclusive that those providing triple-duty care (*P* = 0.0193) experienced more FWE than nonfamily caregivers. Those providing triple-duty care had more FWE than those providing double-duty elder care (*P* = 0.0055).

### Conflict and enrichment prevalence and correlative relationships

Nearly half (47.2%) of participants reported WFC, whereas 14.8% reported FWC. Most experienced WFE (79.4%) and FWE (85.2%). For all participants, higher WFC scores had correlative relationships with higher levels of burnout, lower life satisfaction, and higher intent to quit (Table 2). Higher WFE scores had correlative relationships with lower burnout, higher job satisfaction, higher career satisfaction, higher life satisfaction, and lower intent to quit. There were no practically significant correlative relationships with higher FWC or FWE scores.

**Table 2:** Correlative relationships of conflict and enrichment scores for registered dietitian nutritionists.

	Work-Family Conflict	Family-Work Conflict	Work-Family Enrichment	Family-Work Enrichment
Burnout	.498*	.148*	.428*	.218*
Life Satisfaction	.316*	.247*	.336*	.272*
Job Satisfaction	.294*	.093	.480*	.183*
Career Satisfaction	.257*	.191*	.429*	.209*
Intent to Quit	.303*	.132*	.396*	.159*
Intent to Leave Profession	.256*	.164*	.291*	.113

*Note*: **Bolded** values are considered to be practically significant at the ≥|.30| level.

* Significant at < .0001 level (2-tailed).

### Characteristics related to higher conflict and enrichment scores

This study identified personal and family characteristics contributing to higher WFC/FWC and FWE/WFE scores for RDNs. Data is presented separately for RDNs who held double- or triple-duty care responsibilities and those who did not (nonfamily caregivers).

#### Double- and triple-duty caregiving RDNs

*Conflict*. Individuals with double- or triple-duty care responsibilities who worked <15 hours/week for pay experienced less WFC than those who worked ≥16 hours/week (*P*<0.01; Table 3). Individuals who worked ≥45 hours/week experienced more WFC than those who worked less (*P*<0.001). Individuals with higher self-reported strain had higher WFC (slope = 0.052, *P*<0.001). Individuals in a distressed marriage/partnership experienced more FWC than those who were not (*P* = 0.0011). Those who practiced in non-clinical dietetics areas experienced more FWC than those who practiced in clinical areas (*P* = 0.0009). Individuals who had increased self-reported strain had increased FWC (slope = 0.083, *P*<0.0001).

**Table 3:** Means and Standard Errors (SE) of statistically significant variables with work-family scores based on caregiving status.

	Double- and Triple-Duty Caregiver RDNs^1^				Nonfamily Caregiver RDNs^1^			
	Conflict		Enrichment		Conflict		Enrichment	
	Work-Family	Family-Work	Work-Family	Family-Work	Work-Family	Family-Work	Work-Family	Family-Work
	Mean (SE)	Mean (SE)	Mean (SE)	Mean (SE)	Mean (SE)	Mean (SE)	Mean (SE)	Mean (SE)
**Practice Area**								
Clinical		15.9 (1.20)						
Non-clinical		18.6 (1.40)						
**Avg. hours paid work/week by RDN**								
≤15	14.3 (1.07)				15.2 (2.37)			
16–25	18.7 (0.81)				13.4 (1.36)			
26–35	18.6 (0.72)				15.8 (1.13)			
36–45	20.3 (0.42)				18.0 (0.58)			
>45	24.7 (0.76)				21.9 (0.99)			
**Marital Status**								
Single, never married								
Married/partnered								
Other								
**Marital Satisfaction**								
Not married/partnered		15.5 (2.38)	3.58 (0.11)	3.98 (0.23)	15.2 (0.87)	10.5 (0.45)		3.86 (0.07)
Distressed		19.3 (1.09)	3.66 (0.09)	3.88 (0.06)	18.9 (1.22)	12.8 (0.68)		3.33 (0.10)
Non-distressed		16.9 (1.04)	3.88 (0.07)	4.23 (0.05)	16.5 (0.70)	10.2 (0.35)		4.07 (0.05)
**Mean hours paid work/week by spouse/partner**								
0 or not married/partnered							4.21 (0.10)	
≤15							3.01 (0.39)	
16–25							4.14 (0.32)	
26–35							3.95 (0.22)	
36–45							3.65 (0.15)	
>45							3.92 (0.16)	

^1^ Nonfamily caregivers provide care at work but do not have caregiving responsibilities outside of work; Double-duty and Triple-Duty caregivers provide care at work and provide child and/or elder care outside of work.

*Enrichment*. Individuals with child and/or elder care responsibilities who were not married/partnered experienced less WFE than those in a non-distressed marriage/partnership (*P* = 0.0100). It is suggestive but not conclusive that individuals in a distressed married/partnership experienced less WFE than those in a non-distressed marriage/partnership (*P* = 0.0106). Individuals in a distressed marriage/partnership experienced less FWE than those who were in a non-distressed marriage/domestic partnership (*P*<0.0001).

#### Nonfamily caregiving RDNs

*Conflict*. Nonfamily caregiver RDNs who worked for pay 16–25 hours (*P*<0.001), 26–35 hours (*P* = 0.0004), and 36–45 hours (*P* = 0.0033) per week experienced less WFC that those who worked for pay ≥45 hours/week. It is suggestive but not conclusive that participants in distressed marriages/partnerships had more WFC than those who were not married/partnered (*P* = 0.014). Individuals who were in a distressed marriage/partnership experienced more FWC than those who were not (*P* = 0.0022).

*Enrichment*. Individuals who did not have unpaid caregiving responsibilities and did not have a spouse/partner experienced more WFE than those with a spouse/partner who worked 36–45 hours per week for pay (*P* = 0.0055). Additionally, those who were single/never married experienced less WFE than those who were married/partnered (*P* = 0.0044). Unmarried/partnered individuals experienced more FWE than those in a distressed marriage/partnership (*P*<0.0001). Also, individuals in a distressed marriage/partnership experienced less FWE than those in a non-distressed marriage/partnership (*P*<0.001).

## Discussion

Leaders of the dietetics profession, managers of RDNs, and RDNs can examine these results and respond to the experiences of RDNs. Harnessing the investment in education and practical experience of RDNs while respecting their non-work/family responsibilities is an important effort for maintaining the current dietetics workforce and recruiting others.

### Caregiving

Results from this study show most participants (65.5%) provided either double-duty or triple-duty care which is similar to other studies that found 64% of a female nursing staff [16] and 50% male nursing staff [20] at nursing homes held double- and triple-duty care responsibilities. In a study of physicians, nurses, and other health professionals, 39% of the women (N = 1232) and 41% of the men (N = 174) held double or triple-duty care responsibilities which is notably lower than in this study [40]. Beyond healthcare, in a sample of information technology (IT) professionals (N = 823), 61% reported child and/or elder care responsibilities [41] which is very similar to RDNs in this study. Interestingly, 38% of IT professionals were double-duty child caregivers [41] which is lower than the 47% of RDNs in the present study. In a predominantly female field, knowing many RDNs occupy the role of a double-duty childcare provider is important since a primary reason women leave the workforce in the USA is to stay home with their children [42,43].

The demographic profile of this RDN sample (working, 46.6 years, predominantly female) closely resembles that of someone who provides elder care in the USA which is a 49-year-old woman who works outside the home [44]. A smaller percentage of RDNs (9%) provided double-duty elder care than in the IT professional study (13%) and triple-duty care was about the same (9% vs. 10%) [41]. Employees providing elder care are more likely to present with depression or anxiety, participate in risky health behaviors, and have damaged physical health themselves [45] which all could play a role in poor work performance and employee well-being. Considering RDNs’ unpaid caregiving responsibilities may help managers be effective in determining staffing needs, cross-training strategies, and flexibility with scheduling (e.g., swapping shifts or adjusting start/end times.)

### Conflict

More RDNs reported work conflicting with family (WFC; 47.2%) than family conflicting with work (FWC; 14.8%) which is consistent with findings across the workforce [46] and specifically among nurses [47] and teachers [48–50]. Higher WFC scores had correlative relationships with higher burnout, lower life satisfaction, and higher intent to quit. Burnout is such a significant concern among healthcare professionals that the National Academy of Medicine developed an action collaborative in 2017 to combat burnout and improve clinician well-being [51]. There is limited data about dietitian burnout in the USA, however, in a Canadian study, 57% of dietitians experienced moderate to high burnout [26]. Additionally, an Australian study of hospital-based dietitians experienced lower burnout values than nurses and similar values to the Canadian dietitians [27]. Also interesting is that the more children Australian dietitians had, the less likely they were to experience burnout though this is likely collinear with parttime work [27]. In a study exploring burnout, there was no significant difference in burnout levels between three health professions (speech, physical, and occupational therapy) [52]. Future research could compare RDNs’ work outcomes with other allied health professions, particularly in the context of caregiving, work-family conflict, and work-family enrichment.

Regardless of RDNs’ caregiving responsibilities, those who worked ≥45 hours/week for pay had higher WFC scores than those who worked less. Because RDNs have a finite amount of time, the more time spent at work results in less time for personal or family responsibilities [53]. Others have found that WFC is related to higher turnover and decreased quality of care [54–56]. In nursing, WFC has been identified as a potential barrier to professional entrance and retention [55]. To encourage positive and reduce negative work outcomes, managers may benefit from assessing employee work loads and expectations with the purpose of keeping actual work hours below 45 hours/week. Additionally, it is valuable for managers to maintain the perspective that employees with lower WFC are apt to stay in their positions and avoid burnout as they receive flexible work schedules and obtain adequate paid time off coverage.

Interestingly, few RDNs (14.8%) reported experiencing FWC and there may be several potential explanations for this. Possibilities include individuals who experienced higher WFC may have self-selected out of the workforce, those who have persisted may have established a workable work/family balance, and/or FWC scores may not be accurately reported due to the *family first* mentality which can distort the perception of the actual time and resources used to meet family responsibilities [56,57].

Although this study initially targeted clinical RDNs, data from RDNs practicing in non-clinical roles was captured. Non-clinical RDNs with caregiving responsibilities experienced higher FWC than clinical RDNs. Further research should explore how RDNs in various practice areas experience the work/family interface in an effort to help students and RDNs make career decisions that align with their values and other responsibilities.

### Enrichment

Encouragingly, most RDNs (79.4%) self-reported experiencing WFE. Those who experienced higher levels of WFE also experienced lower burnout, higher job satisfaction, higher career satisfaction, and lower intent to quit. In the literature, there seems to be a stronger relationship between enrichment scores and job satisfaction when samples have more women, which is the case in the present study [25]. There is also evidence that national culture influences the relationship between WFE and job satisfaction [58], thus it would be beneficial to explore this relationship with dietitians from different countries. Overwhelmingly, RDNs (85.2%) indicated that their family life enriched their work life and showcasing this can further the family-friendly reputation the dietetics profession holds. University advisors can share this information with students considering the profession and managers can see employees’ family lives as assets to work. Professional leadership, educators, and managers can emphasize these findings during recruitment.

For RDNs, marital/partnership status and quality (distressed or non-distressed) appear to be associated with how they experience WFE and FWE. This is consistent with a work-family enrichment meta-analysis that revealed those who were married/partnered typically report higher FWE [59]. Further, other research has found that WFE and FWE were positively associated with marital satisfaction [60]. Managers can promote employee assistance programs or other employee benefits that make individual and/or couples counseling accessible and/or affordable. It is important to recognize that both work and family do provide sources of enrichment that enhance the positive traits and performance required for work and personal/family life [23].

Much of the research about caregiving roles, work-family conflict, and work-family enrichment of healthcare professions has focused on nurses. Though dietetics is also predominantly female, and the work settings are similar, a dietitian’s work may differ in physical tasks and other ways. This study may provide a foundation for other predominantly female professions particularly in healthcare to explore and compare the work/life interface of practitioners.

Self-reporting may be a limitation to this study as responses may have been biased (e.g., not reporting high levels of FWC). The nature of the measures used reflected experiences RDNs had within six months of the study and did not capture each phase of participants’ lives/careers. Further research should explore these issues internationally as work/family policies differ across countries; differences across dietetics practice areas and responsibility levels; and strategies (organizational or personal) that RDNs have found helpful for their work/life interface.

## Conclusion

Showcasing the high percentage of RDNs who experienced WFE/FWE may assist in attraction and retention efforts for the dietetics profession. In addition, findings suggest that hours worked may have a more direct impact on WFC than caregiving responsibilities, although this needs further research. Managers of RDNs may benefit from discovering opportunities to reduce WFC/FWC and assisting their employees in the interplay of work and family.

## Supporting information

S1 File Work/Family conflict & caregiving. Emily Patten and Karla Williams collected this data in the spring of 2018.
(DOCX)
