# Supplementary material for: How work and family caregiving responsibilities interplay and affect registered dietitian nutritionists and their work: A national survey
Source: PLoS One. 2021 Mar 10;16(3):e0248109. doi: 10.1371/journal.pone.0248109 (PMC7946290; doi:10.1371/journal.pone.0248109)
Supplement: S1 File — Emily Patten and Karla Williams collected this data in the spring of 2018. (DOCX) [file pone.0248109.s001.docx]

Work/Family Conflict & Caregiving

Emily Patten and Karla Williams collected this data in the spring of 2018

Start of Block: Initial

| \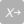 |
| --- |

Q2 What best describes your current employment situation?

1. Employed as Registered Dietitian Nutritionist (RDN) (1)
2. Employed in a field other than dietetics (3)
3. Not employed, looking for work (4)
4. Not employed, NOT looking for work (5)
5. Disabled, not able to work (6)
6. Retired (7)
7. Other: (8) ________________________________________________

Display This Question:

If Q2 != Employed as Registered Dietitian Nutritionist (RDN)

Q3 Do you plan on re-entering the **dietetics** workforce at some point?

1. No, never (1)
2. Yes, within 1 year (6)
3. Yes, in 1-3 years (2)
4. Yes, in 4-6 years (3)
5. Yes, in 7-9 years (4)
6. Yes, 10+ years (5)

| Page Break |  |
| --- | --- |

End of Block: Initial

Start of Block: Family Characteristics/Caregiving

Q6 Are you a parent, step parent, or legal guardian of a child or children who live with you full-time or part-time?

1. Yes (1)
2. No (2)

| Page Break |  |
| --- | --- |

Display This Question:

If Q6 = Yes

Q7 How many children live with you full-time or part-time?

▼ 1 (1) ... 13+ (13)

| Page Break |  |
| --- | --- |

Display This Question:

If Q6 = Yes

Q8 What is the age of the youngest child living with you? (Indicate age in years or "infant" if under 1)

________________________________________________________________

| Page Break |  |
| --- | --- |

Q9 Elder care is defined as providing unpaid care (e.g., managing a person's finances, arranging for outside services, visiting regularly to see how they are doing, helping with personal needs, or household chores) for at least 3 hours per week to an older adult, regardless of their living arrangement.   Have you provided elder care over the past 6 months?

1. Yes (1)
2. No (2)

| Page Break |  |
| --- | --- |

Display This Question:

If Q6 = Yes

Or Q9 = Yes

Q10 Please drag the indicator on the scale to how burdensome you feel your **unpaid** child or elder caregiving responsibilities (either family or non-family) have been over the past 6 months.    For the scale, ‘0’ means that you feel that your unpaid caregiving responsibilities were not straining at all; ‘100’ means that you feel they were much too straining.

|  | Not at all straining | Much too straining | Not Applicable |
| --- | --- | --- | --- |

|  | 0 | 10 | 20 | 30 | 40 | 50 | 60 | 70 | 80 | 90 | 100 |
| --- | --- | --- | --- | --- | --- | --- | --- | --- | --- | --- | --- |

| Level of strain () | 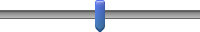 |
| --- | --- |

| Page Break |  |
| --- | --- |

Q11 What is your current marital status?

1. Single, never married (1)
2. Married or domestic partnership (2)
3. Widowed (3)
4. Divorced (4)
5. Separated (5)
6. Other, please indicate: (6) ________________________________________________

| Page Break |  |
| --- | --- |

Display This Question:

If Q11 = Married or domestic partnership

Q14 You indicated that you have a spouse/partner.  Please rate your satisfaction:

|  | Extremely Dissatisfied (1) | Very Dissatisfied (2) | Somewhat Dissatisfied (3) | Mixed (4) | Somewhat Satisfied (5) | Very Satisfied (6) | Extremely Satisfied (7) |
| --- | --- | --- | --- | --- | --- | --- | --- |
| How satisfied are you with your marriage/ partnership? (1) |  |  |  |  |  |  |  |
| How satisfied are you with your partner as a spouse/ partner? (2) |  |  |  |  |  |  |  |
| How satisfied are you with your relationship with your spouse/ partner? (3) |  |  |  |  |  |  |  |

Q15 Please think about your life as a whole, how satisfied are you with it?

1. Completely satisfied (1)
2. Very satisfied (2)
3. Somewhat satisfied (3)
4. Not very satisfied (4)
5. Not at all satisfied (5)

| Page Break |  |
| --- | --- |

Q18 **There are diverse types of families, please respond to the following questions based on what *you* consider to be *your* family.**

Q19 Read each statement below and mark how strongly you agree or disagree.

|  | Strongly disagree (1) | Disagree (2) | Somewhat disagree (3) | Neither agree nor disagree (4) | Somewhat agree (5) | Agree (6) | Strongly Agree (7) |
| --- | --- | --- | --- | --- | --- | --- | --- |
| The demands of my work interfere with my home and family life. (1) |  |  |  |  |  |  |  |
| The amount of time my job takes up makes it difficult to fulfill family responsibilities. (2) |  |  |  |  |  |  |  |
| Things I want to do at home do not get done because of the demands my job puts on me. (3) |  |  |  |  |  |  |  |
| My job produces strain that make it difficult to fulfill family duties. (4) |  |  |  |  |  |  |  |
| Due to work-related duties, I have to make changes to my plans for family activities. (5) |  |  |  |  |  |  |  |

| Page Break |  |
| --- | --- |

Q20 Read each statement below and mark how strongly you agree or disagree.

|  | Strongly disagree (1) | Disagree (2) | Somewhat disagree (3) | Neither agree nor disagree (4) | Somewhat agree (5) | Agree (6) | Strongly Agree (7) |
| --- | --- | --- | --- | --- | --- | --- | --- |
| The demands of my family or spouse/partner interfere with work-related activities. (6) |  |  |  |  |  |  |  |
| I have to put off doing things at work because of demands on my time at home. (7) |  |  |  |  |  |  |  |
| Things I want to do at work don't get done because of the demands of my family or spouse/partner. (8) |  |  |  |  |  |  |  |
| My home life interferes with my responsibilities at work such as getting to work on time, accomplishing daily tasks, and working overtime. (9) |  |  |  |  |  |  |  |
| Family-related strain interferes with my ability to perform job-related duties. (10) |  |  |  |  |  |  |  |

| Page Break |  |
| --- | --- |

Q21 Please rate your level of agreement for the following statements:

 **My involvement in my work...**

|  | Strongly disagree (1) | Somewhat disagree (2) | Neither agree nor disagree (3) | Somewhat agree (4) | Strongly agree (5) |
| --- | --- | --- | --- | --- | --- |
| Helps me to understand different viewpoints and this helps me be a better family member. (1) |  |  |  |  |  |
| Makes me feel happy and this helps me be a better family member. (2) |  |  |  |  |  |
| Helps me feel personally fulfilled and this helps me be a better family member. (3) |  |  |  |  |  |

| Page Break |  |
| --- | --- |

Q22 Please rate your level of agreement for the following statements:

 My involvement in my family...

|  | Strongly disagree (1) | Somewhat disagree (2) | Neither agree nor disagree (3) | Somewhat agree (4) | Strongly agree (5) |
| --- | --- | --- | --- | --- | --- |
| Helps me acquire skills and this helps me be a better worker. (1) |  |  |  |  |  |
| Puts me in a good mood and this helps me be a better worker. (2) |  |  |  |  |  |
| Encourages me to use my work time in a focused manner and this helps me be a better worker. (3) |  |  |  |  |  |

| Page Break |  |
| --- | --- |

End of Block: Work-Life Balance

Start of Block: Burnout, Satisfaction, and Intent to Quit

Q23 The balance between my personal and professional commitments is about right.

1. Strongly agree (8)
2. Agree (9)
3. Neither agree nor disagree (10)
4. Disagree (11)
5. Strongly disagree (12)

Q24 I am currently experiencing burnout in my work.

1. Strongly agree (8)
2. Agree (9)
3. Neither agree nor disagree (10)
4. Disagree (11)
5. Strongly disagree (12)

| Page Break |  |
| --- | --- |

Q25 How do you feel about your present job in general?

1. Very satisfied (1)
2. Satisfied (2)
3. Neither satisfied nor dissatisfied (3)
4. Dissatisfied (4)
5. Very dissatisfied (5)

Q26 All things considered, I am satisfied with my career as a dietitian.

1. Strongly agree (15)
2. Agree (16)
3. Neither agree nor disagree (17)
4. Disagree (18)
5. Strongly disagree (19)

| Page Break |  |
| --- | --- |

Q27 Read each statement below and mark how strongly you agree or disagree with each statement.

|  | Strongly disagree (1) | Disagree (2) | Somewhat disagree (3) | Neither agree nor disagree (4) | Somewhat agree (5) | Agree (6) | Strongly agree (7) |
| --- | --- | --- | --- | --- | --- | --- | --- |
| As soon as I can find a better job, I'll quit. (1) |  |  |  |  |  |  |  |
| I often think about quitting my job. (2) |  |  |  |  |  |  |  |

Q28 How often in the course of the past year have you thought about giving up dietetics completely and starting a different kind of profession?

1. Never (1)
2. A few times (2)
3. A few times a month (3)
4. A few times a week (4)
5. Every day (5)

| Page Break |  |
| --- | --- |

End of Block: Burnout, Satisfaction, and Intent to Quit

Start of Block: Work place support

Start of Block: Professional and Work Characteristics

Q32 What best describes your **primary** practice area within dietetics?

1. Clinical nutrition - Acute care/inpatient (1)
2. Clinical nutrition - Ambulatory care/outpatient (2)
3. Clinical nutrition - Long-term care (3)
4. Clinical nutrition - Home health care (4)
5. Community (6)
6. Food and nutrition management (7)
7. Consultation and business (8)
8. Education and research (9)
9. Other (please indicate): (5) ________________________________________________

Q33 How many paying jobs do you currently have?

▼ 1 (1) ... 10+ (10)

Q34 Over the past 6 months, on average, how many hours have **you** worked for pay each week?
 
Please indicate hours using digits and round to the nearest hour.

________________________________________________________________

| Page Break |  |
| --- | --- |

Q35 How many years have you been a RDN?

1. Less than 1 year (1)
2. 1-5 years (7)
3. 6 -10 years (2)
4. 11-15 years (3)
5. 16-20 years (4)
6. 21-25 years (5)
7. 26 or more years (6)

Q36 How many years have you had your **present job**?

1. Less than 1 year (1)
2. 1-5 years (8)
3. 6-10 years (2)
4. 11-15 years (3)
5. 16-20 years (4)
6. 21-25 years (6)
7. 26 or more years (7)

| Page Break |  |
| --- | --- |

Q37 Is your primary supervisor a Registered Dietitian Nutritionist?

1. Yes (1)
2. No (2)
3. Not Applicable (3)

Q38 Which of the following best describes your employer?

1. Contract Management Company (1)
2. Self-Operated Organization (2)
3. Self-Employed (3)

| Page Break |  |
| --- | --- |

Q39 How would you self-assess your level of professional involvement in the past 3 years?  


Consider your involvement in local, state, or national levels of professional organizations like the Academy of Nutrition and Dietetics, American Society for Parenteral and Enteral Nutrition, American Diabetes Association, etc.

1. Not involved (1)
2. Somewhat involved (2)
3. Involved (3)
4. Very involved (4)

Q40 Are you currently a member of the Academy of Nutrition and Dietetics?

1. Yes (1)
2. No, but I have been in the past (2)
3. No, I never have been (3)

End of Block: Professional and Work Characteristics

Start of Block: General Characteristics

Display This Question:

If Q11 = Married or domestic partnership

Q41 Does your spouse/partner work for pay?

1. Yes (1)
2. No (2)

| Page Break |  |
| --- | --- |

Display This Question:

If Q41 = Yes

Q42 Over the past 6 months, on average, how many hours has your **spouse/partner** worked for pay each week?
 
Please indicate hours using digits and round to the nearest hour.

________________________________________________________________

Q43 For the past 12 months, what was your household's income from all sources?

1. Less than $30,000 (1)
2. $30,000 - $39,999 (3)
3. $40,000 - $49,999 (4)
4. $50,000 - $59,999 (5)
5. $60,000 - $69,999 (6)
6. $70,000 - $79,999 (7)
7. $80,000 - $89,999 (8)
8. $90,000 - $99,999 (9)
9. $100,000 - $149,999 (10)
10. More than $150,000 (11)
11. Prefer not to answer (12)

| Page Break |  |
| --- | --- |

Display This Question:

If Q11 = Married or domestic partnership

Q44 Who makes more money?

1. My spouse/partner (1)
2. Me (2)
3. We make about the same (3)
4. Prefer not to answer (4)

| Page Break |  |
| --- | --- |

Q45 With which gender do you identify?

1. Female (1)
2. Male (2)
3. Other (please indicate): (3) ________________________________________________
4. Prefer not to answer (4)

Q46 What is your highest level of education earned?

1. Bachelor's degree (1)
2. Master's degree (2)
3. Doctorate degree (3)

Q47 What is your age in years?

________________________________________________________________

Q48 Where do you currently reside?

▼ Alabama (1) ... I do not reside in the United States (53)

| Page Break |  |
| --- | --- |

Q49 Are you Hispanic, Latino/a, or Spanish origin?

1. Yes (1)
2. No (2)
3. Prefer not to answer (3)

Q50 What is your race?

1. White (1)
2. Black or African American (2)
3. American Indian or Alaska Native (3)
4. Asian (4)
5. Native Hawaiian or Pacific Islander (5)
6. Other (please indicate): (6) ________________________________________________
7. Prefer not to answer (7)

| Page Break |  |
| --- | --- |

References

1. National Alliance for Caregivers, AARP Public Policy Institute Caregiving in the U.S. NAC and AARP Public Policy Institute; 2015 [cited 2021 Jan 25]. Available from: <https://www.aarp.org/content/dam/aarp/ppi/2015/caregiving-in-the-united-states-2015-report-revised.pdf>
2. DePasquale N, Davis KD, Zarit SH, Moen P, Hammer LB, Almeida DM. Combining formal and informal caregiving roles: The psychosocial implications of double- and triple-duty care. J Gerontol B Psychol Sci Soc Sci. 2016;71:201-11.
3. Netemeyer RG, Boles JS & McMurrian R. Development and validation of work–family conflict and family–work conflict scales. J App Psychol. 1996;81:400-10. 21
4. Kacmar KM, Crawford WS, Carlson DS, Ferguson M, Whitten D. A short and valid measure of work-family enrichment. J Occup Health Psychol. 2014;19:2-45.
5. Schumm WR, Paff-Bergen LA, Hatch RC, Obiorah FC, Copeland JM, Meens LD, Bugaighis MA. Concurrent and discriminant validity of the Kansas marital satisfaction scale. J Marriage Fam. 1986:381-7.
6. van Exel NJ, Brouwer WB, van den Berg B, Koopmanschap MA, van den Bos GA. What really matters: An inquiry into the relative importance of dimensions of informal caregiver burden. Clin Rehabil. 2004;18:683-93.
7. Starmer AJ, Frintner MP & Freed GL. Work–life balance, burnout, and satisfaction of early career pediatricians. Pediatrics, 2016;137: e20153183.
8. Scarpello V, Campbell JP. Job satisfaction: are all the parts there? Personnel Psychol. 1983;36:577-600.
9. Begley TM & Czajka JM. Panel analysis of the moderating effects of commitment on job satisfaction, intent to quit, and health following organizational change. J Appl Psychol. 1993;78:552-6.
10. Hasselhorn H, Tackenberg P & Müller B. NEXT-Study Group. Working conditions and intent to leave the profession among nursing staff in Europe. Stockholm, Sweden: National Institute for Working Life Stockholm; 2003. Report No 7:2003.
